# Local Spatial–Temporal Matching Method for Space-Based Infrared Aerial Target Detection

**DOI:** 10.3390/s22051707

**Published:** 2022-02-22

**Authors:** Lue Chen, Peng Rao, Xin Chen, Maotong Huang

**Affiliations:** 1 Key Laboratory of Intelligent Infrared Perception, Chinese Academy of Sciences, Shanghai 200083, China; chenlue@mail.sitp.ac.cn (L.C.); chenxin@mail.sitp.ac.cn (X.C.); 2 Shanghai Institute of Technical Physics, Chinese Academy of Sciences, Shanghai 200083, China; 3 University of Chinese Academy of Sciences, Beijing 100049, China

**Keywords:** space-based infrared detector, aerial target detection, local spatial–temporal matching, staring imaging mode

## Abstract

The feature of a space-based infrared signal is that the intensity of clutter is much stronger than that of an aerial target. Such a feature poses a great challenge to aerial target detection since the existing infrared target detection methods are prone to enhance clutter but ignore the real target, which results in missed detection and false alarms. To tackle the challenge, we propose a concise method based on local spatial–temporal matching (LSM). Specifically, LSM mainly consists of local normalization, local direction matching, spatial–temporal joint model, and inverse matching. Local normalization aims to enhance the target to the same strength as the clutter, so that the weak target will not be ignored. After normalization, a direction-matching step is applied to estimate the moving direction of the background between the basic frame and referenced frame. Then the spatial–temporal joint model is constructed to enhance the target and suppress strong clutter. Similarly, inverse matching is conducted to further enhance the target. Finally, a salience map is obtained, on which the aerial target is extracted by the adaptive threshold segmentation. Experiments conducted on four space-based infrared datasets indicate that LSM handles the above challenge and outperforms seven state-of-the-art methods in space-based infrared aerial target detection.

## 1. Introduction

The task of aerial target detection is of great importance in many fields, including air traffic surveillance [1] and intelligence reconnaissance [2]. Space-based infrared (IR) imaging technology has the advantages of all-day and wide-area imaging, while both the on-orbit experiment [3] and ground theoretical research [4] have certified that an aerial target can be detected by space-based IR detectors. Therefore, research on space-based IR aerial target detection continues to attract much attention.

**Figure 1:**
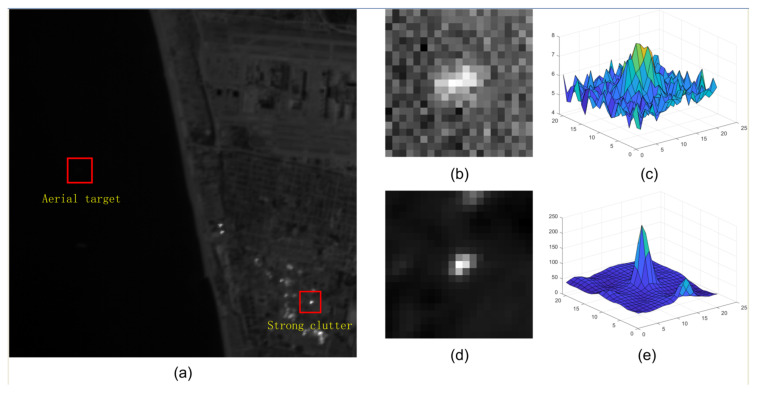
(**a**) The aerial target and strong clutter in the space-based IR image; their details are magnified in (**b**–**e**). (**b**) The local slice of an aerial target. (**c**) Three-view of the aerial target. (**d**) Local slice of strong clutter. (**e**) Three-view of strong clutter.

The sizes of the aerial targets on the space-based IR images range from 5 × 5 to 9 × 9 pixels, which is in accordance with the definition [5] of IR small target. However, space-based IR aerial target detection is considerably different from ground-based IR small target detection. First, a remote imaging distance (>300 km) weakens the intensity of aerial targets. Second, the complex earth background and frequent human activities generate strong clutter whose spatial characters are similar to the small target in space-based images. The above two factors result in a feature in which the aerial target is much weaker than the clutter in the space-based IR image. As shown in Figure 1, both aerial target and clutter cover several pixels in the space-based IR image, while the intensity of clutter is nearly twenty times stronger than that of the target. During detection, the weak target may lead to missed detection, and the strong clutter could yield false alarms. Third, the computational resources are limited on the space-based platform, while they are unlimited on the ground-based platform, which means the space-based detection method must be resource-friendly. Therefore, how to enhance weak targets and suppress strong clutter efficiently has been a critical issue for the space-based detection method, and it has been the challenge for space-based IR aerial target detection.

In the past decades, researchers continually proposed hundreds of IR small target detection methods, aiming for efficient detection under different scenarios. Nevertheless, most methods are proposed for ground-based detection instead of space-based detection.

The filter-based methods, such as TDLMS [6] and Top-hat [7], are easy to achieve, but they still struggle to enhance weak the target. Recently, some researchers have designed more complicated filters to detect small targets under specific background; for example, Lu et al. proposed a filter-based method for maritime IR small target detection [8]. The local contrast method (LCM) proposed by Chen et al. [9] attracts much attention for the concise structure. Additionally, a great number of LCM-based methods [10,11,12] working on the ground-based platform have subsequently been proposed and detect small targets under complex backgrounds. Moreover, most space-based detection methods, such as local blob-like contrast map and local gradient map (LBCM-LGM) [13], neighborhood saliency map (NSM) [14], spatial–temporal local contrast method (STLCM) [2], and spatial–temporal local contrast filter (STLCF) [15], are LCM based. Though these methods perform well on weak target enhancement, they suppress the strong clutter inefficiently, leading to false alarms. In recent years, the mainstream detection methods are mostly based on IR image patch (IPI), low-rank representation (LLR), and deep learning (DL). According to the different spatial correlations of target and background, some methods based on IPI [16,17,18,19] or LLR [20,21,22] have been proposed to extract the small target from the IR image. However, these methods cannot distinguish the real target from the background when the target has close intensity to its neighboring region. In addition, they show poor real-time performance since they require a great number of iterations during optimization. Other mainstream methods based on DL [23,24,25,26] show effective performance in complex backgrounds. However, the performance of DL-based methods severely relies on their datasets; thus, they are not suitable for space-based detection because the space-based dataset is scarce in its current state.

As far as we know, although thousands of infrared small target detection methods have been proposed, most are ground-based detection methods; the number of space-based detection methods is much fewer than ground-based methods. Most existing space-based detection methods are LCM-based since computational resources on space platforms are limited while LCM-based methods are easy to implement on hardware and consume fewer computational resources than IPI- or DL-based methods. In 2018, a single-frame method called neighboring saliency map (NSM) for space-based detection was proposed and detected a dim target with a signal-to-clutter (*SCR*) less than 1. The space-based detection methods based on spatial–temporal local contrast maps (STLCF) [15] and spatial–temporal local contrast maps (STLCM) [2] are both LCM-based methods. Lv et al. proposed a method that detects the space-based weak moving target with an *SCR* ≈1 or even <1 [27]. They further proposed a dim small moving target detection and tracking method based on a spatial–temporal joint processing model (STJP) [28], which also performed well on space-based dim target detection. However, the existing space-based detection methods mainly focus on dim target enhancement but ignore interference resulting from the strong clutter.

Although current methods, both space-based and ground-based methods, achieve detection in complex backgrounds, they are only for conditions where the target strength is close to or stronger than the clutter or highly light background. Therefore, it is significant to overcome the space-based detection challenge posed by a feature in which the clutter is much stronger than the aerial target.

To conquer the above challenge, we propose a space-based IR aerial target detection method based on local spatial–temporal matching (LSM), which has a concise structure. The contributions of LSM are given as follows.

(1) Local normalization is proposed to shorten the difference between aerial target and strong clutter, which ensures that the weak target and strong clutter will be processed in subsequent steps within the same value domain.
(2) Local direction matching and spatial–temporal joint model are constructed to suppress the strong clutter and enhance aerial target by considering the spatial–temporal difference between aerial target and background.
(3) A reverse matching step is leveraged to further enhance the target and eliminate the residual clutter.
(4) Experiments conducted on the space-based IR datasets demonstrate that LSM can enhance the weak target and suppress the strong clutter simultaneously and effectively and that it performs better than the existing methods on space-based IR aerial target detection.

## 2. Proposed Methods

**Figure 2:**
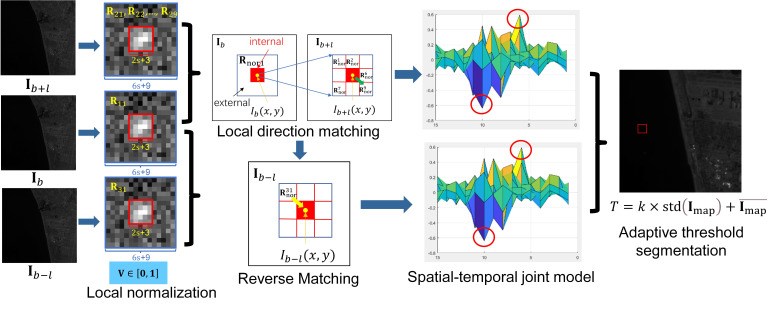
Overview of LSM.

The local spatial–temporal matching detection method (LSM) is suitable for the IR image sequence obtained by a space-based platform under staring imaging mode. LSM consists of five steps: local normalization, local direction matching, spatial–temporal joint model, reverse matching, and adaptive threshold segmentation. The details of LSM are elaborated in this section, and an overview is given in Figure 2.

### 2.1. Local Slices Extraction and Normalization

**Figure 3:**
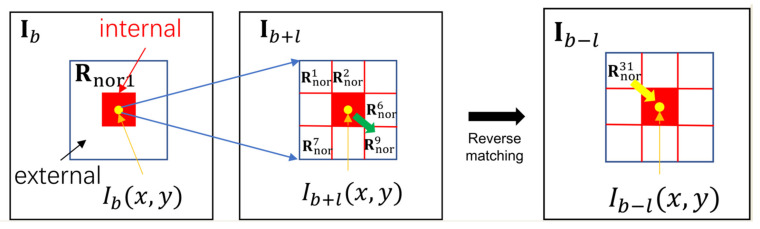
Local spatial–temporal matching and reverse matching.

**Figure 4:**
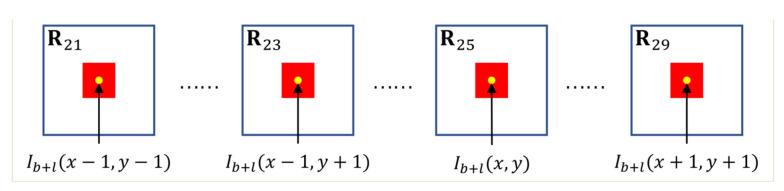
Positions of the local slices in the reference frame $I_{b+l}$.

The first step in the proposed methods is local normalization, which reduces the sensitivity of the subsequent steps to the strong clutter. As shown in Figure 2, at the local normalization step, a local slice named $R_{11}$ is extracted at the point $\left(x,y\right)$ in the base frame $I_{b}$ (b represents the frame number in the sequence). The neighboring region of $R_{11}$ is defined in Equation (1):

(1) $$\Omega_{R_{11}}=\left\{\left(i,j\right)|\max\left(\left|i-x\right|,\left|j-y\right|\right)\leq 3s+4\right\},s=1,\;2,\;3,\;4,$$

where $\Omega_{R_{11}}$ is the neighboring region, and s is the radius of the target. In reference frame $I_{b+l}$, the local region of the pixel $I_{b+l}\left(x,y\right)$ represented by $\Omega_{\mathrm{local}}$ is extracted and defined as:

(2) $$\Omega_{\mathrm{local}}=\left\{\left(p,q\right)|\max\left(\left|p-x\right|,\left|q-y\right|\right)\leq r_{\mathrm{match}}\right\},$$

where l represents frame interval, and $r_{\mathrm{match}}$ represents the matching radius determined by practical engineering tasks. In our work, $r_{\mathrm{match}}$ is set to 1; the range of is illustrated in Figure 2 and Figure 3. Then nine slices with the same dimension as $R_{11}$ are extracted and named $R_{2m},m=1,2,3,\ldots ,9$. The positions of $R_{2m}$s are further illustrated in Figure 4, and the yellow points are the centers of $R_{2m}$s.

The considerable difference between target and strong clutter causes missed detection and false alarm. Thus, the local normalization is designed to transfer the intensity into range $\left[0,1\right]$. The definition of local normalization is as follows:

(3) Rnor1g,h=R11g,h−minR11maxR11−minR11,

(4) Rnormg,h=R2mg,h−minR2mmaxR2m−minR2m,

where $\left(g,h\right)$ represents the position in the $R_{11}$ and $R_{2m}$s, $R_{\mathrm{nor}1}\left(g,h\right)$ means the normalized value at the point $\left(g,h\right)$ within $R_{11}$, $R_{\mathrm{nor}}^{m}\left(g,h\right)$ does the same. After the local normalization, both the target and clutter are processed within the value domain $\left[0,1\right]$ during the subsequent steps.

### 2.2. Local Direction Matching

When the space-based imaging system works under the staring mode, the backgrounds including the strong clutter in the IR sequence are moving within a tiny area. Therefore, the background can be supposed to move straightly in a short frame interval. Local direction matching is designed to determine which local slice of $R_{2m}$ in $I_{b+l}$ is the most similar to $R_{11}$ in the $I_{b}$. In this paper, the local matching function is designed to measure the matching degree. The matching coefficient at point $\left(x,y\right)$ is also determined. The functions are given as follows:

(5) $$r_{m}=\frac{2\times \sum_{g,h}\left[\left|R_{\mathrm{nor}1}\left(g,h\right)-\bar{R_{\mathrm{nor}1}}\right|\times \left|R_{\mathrm{nor}}^{m}\left(g,h\right)-\bar{R_{\mathrm{nor}}^{m}}\right|\right]}{\sum_{g,h}{\left(R_{\mathrm{nor}1}\left(g,h\right)-\bar{R_{\mathrm{nor}1}}\right)}^{2}+\sum_{g,h}{\left(R_{\mathrm{nor}}^{m}\left(g,h\right)-\bar{R_{\mathrm{nor}}^{m}}\right)}^{2}},$$

(6) $$r_{1}\left(x,y\right)=\max\left(r_{m}\right),$$

(7) $$m_{\max}=\underset{m}{\arg\;\max}r_{m},$$

where $r_{m}$s represents the matching degree between $R_{\mathrm{nor}1}$ and $R_{\mathrm{nor}}^{m}$s, $r_{1}$ is the matching coefficient, and $m_{\max}$ determines the local slice in $R_{\mathrm{nor}}^{m}$s that is most similar to $R_{\mathrm{nor}1}$. As shown in Figure 3, if $m_{\max}=9$, $R_{\mathrm{nor}}^{9}$ is the slice matching to $R_{\mathrm{nor}1}$, which means the background moves from $\left(x,y\right)$ to $\left(x+1,y+1\right)$ during $\left[b,\;b+l\right]$, as illustrated by the green arrow at the local direction matching step.

### 2.3. Spatial–Temporal Joint Model

Once local direction matching is performed, suppression of strong clutter and aerial target enhancement can be achieved by the spatial–temporal joint model. First, the difference slice $R_{\mathrm{dif}}$ is obtained by local slice difference:

(8) $$R_{\mathrm{dif}}=R_{\mathrm{nor}1}-R_{\mathrm{nor}}^{m_{\max}},$$

after which most backgrounds, including the clutter in $I_{b}$, are suppressed initially, even if the clutter is much stronger than the aerial target.

The neighboring region of $R_{\mathrm{dif}}$ is divided into internal and external regions, and their relationships are given as follows:

(9) $$\Omega_{\mathrm{int}}=\left\{\left(g,h\right)|\max\left(\left|g-x\right|,\left|h-j\right|\right)\leq s+1\right\},s=1,2,3,$$

(10) $$\Omega_{\mathrm{int}}\cup \Omega_{\mathrm{ext}}=\Omega_{R_{\mathrm{dif}}},$$

(11) $$\Omega_{\mathrm{int}}\cap \Omega_{\mathrm{ext}}=\emptyset ,$$

where $\Omega_{\mathrm{int}}$ and $\Omega_{\mathrm{ext}}$ are the internal and external regions, respectively, and $\Omega_{R_{\mathrm{dif}}}$ represents the neighboring region of $R_{\mathrm{dif}}$, which has the same range as $\Omega_{R_{11}}$; ∅ is the null set. The relationship between $\Omega_{\mathrm{int}}$ and $\Omega_{\mathrm{ext}}$ is illustrated in Figure 3, where the red region represents $\Omega_{\mathrm{int}}$, and the rest of the blue rectangle represents the range of $\Omega_{\mathrm{ext}}$.

Then, the nonuniformity stripes resulting from the inadequate preprocessing can be suppressed by the equation:

(12) $$d_{\mathrm{dif}1}\left(x,y\right)=\max\left(R_{\mathrm{int}}\right)-\max\left(R_{\mathrm{ext}}\right),$$

where $R_{\mathrm{int}}$ is the matrix constructed by the pixels in $\Omega_{\mathrm{int}}$, and $R_{\mathrm{ext}}$ does the same.

If the target appears, the dipole containing positive and negative peaks are left in $R_{\mathrm{int}}$. The dipole is highlighted by a pair of red circles in Figure 2. Thus, the dipole value at $\left(x,y\right)$ is extracted:

(13) $$d_{\mathrm{dipole}1}\left(x,y\right)={\left[\max\left(R_{\mathrm{int}}\right)-\min\left(R_{\mathrm{int}}\right)\right]}^{2},$$

where $d_{\mathrm{dipole}1}$ is the dipole value. At this step, the clutter can be further suppressed, but the aerial target can be significantly enhanced by quadratic operation.

Finally, the value of local spatial–temporal matching between $I_{b}$ and $I_{b+l}$ is calculated as:

(14) $$I_{v1}\left(x,y\right)=\left[1-r_{1}\left(x,y\right)\right]\times d_{\mathrm{dif}1}\left(x,y\right)\times d_{\mathrm{dipole}1}\left(x,y\right),\;$$

where $I_{v1}\left(x,y\right)$ represents the matching value at the point $\left(x,y\right)$.

### 2.4. Reverse Matching

After obtaining $I_{v1}\left(x,y\right)$, reverse matching is added into LSM. As shown in Figure 3, at $\left(x,y\right)$, if the offset from $I_{b}$ and $I_{b+l}$ is in the direction indicated by the green arrow, the offset from $I_{b-l}$ and $I_{b}$ is in the direction indicated by the yellow arrow in Figure 3, and $I_{b-l}$ is another reference frame. In this case, the local backgrounds in $I_{b-l}$ and $I_{b}$ can be reverse-matched. At $\left(x,y\right)$ in $I_{b}$, the offsets of local background from $I_{b-l}$ to $I_{b}$ can be determined by:

(15) $$dy=\left\{\begin{array}{c} +1,\;m_{\max}=3,6,9\;\\ 0,m_{\max}=2,5,8\\ -1,\;m_{\max}=1,4,7 \end{array}\right.,$$

(16) dx=fix9−mmax3−1,

where dy and dx denote the offsets in the horizontal and vertical directions, respectively, and $\mathrm{fix}\left(*\right)$ represents the operation calculating the nearest integer in the direction to zero.

As shown in Figure 3, the local slice in $I_{b-l}$, given by $R_{31}$, is determined in the reverse matching step. The neighboring region of $R_{31}$ is formulated as:

(17) $$\Omega_{R_{31}}=\left\{\left(i,j\right)|\max\left(\left|x+dx-i\right|,\left|y+dy-j\right|\right)\leq 3\times s+4\right\},\;$$

where $\Omega_{R_{31}}$ denotes the neighboring region of $R_{31}$. The normalized slice of $R_{31}$ and the matching coefficient between $R_{11}$ and $R_{31}$ are obtained by Equations (3) and (5), and $R_{\mathrm{nor}}^{31}$ and $r_{2}\left(x,y\right)$ represent the normalized slice and matching coefficient, respectively.

Finally, the spatial–temporal joint model is constructed. Identically, the value of local spatial–temporal matching between $I_{b}$ and $I_{b-l}$ is calculated by Equations (8)–(14) and represented by $I_{v2}\left(x,y\right)$.

### 2.5. Adaptive Threshold Segmentation

**Figure 5:**
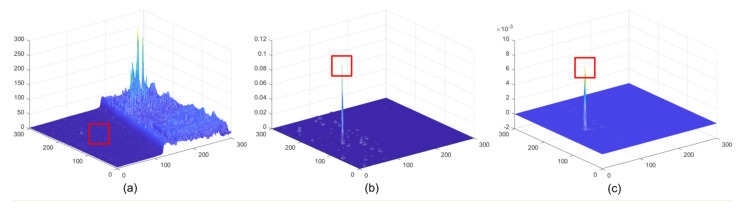
(**a**) Three-view of raw image; (**b**) three-view of the result after spatial–temporal matching; (**c**) three-view of the saliency map.

The mean filter is introduced to suppress noise, which is conducted as:

(18) $$d_{\mathrm{dif}2}\left(x,y\right)=R_{\mathrm{nor}1}\left(3\times s+5,3\times s+5\right)-\bar{R_{\mathrm{nor}1}},\;$$

where $d_{\mathrm{dif}2}\left(x,y\right)$ denotes the value after mean filtering at point $\left(x,y\right)$. In addition, the saliency map $I_{\mathrm{map}}$ is obtained by:

(19) $$I_{\mathrm{map}}\left(x,y\right)=d_{\mathrm{dif}2}\left(x,y\right)\times I_{v1}\left(x,y\right)\times I_{v2}\left(x,y\right),\;$$

where $I_{\mathrm{map}}\left(x,y\right)$ is the map value at $\left(x,y\right)$. The results are shown in Figure 5; even though the target is much weaker than clutter in Figure 1 and Figure 5a, it is enhanced significantly, and the strong clutter is well suppressed.

In $I_{\mathrm{map}}$, clutter and background are suppressed, but the IR aerial target is retained and enhanced. Finally, the aerial target is detected by adaptive threshold segmentation:

(20) $$T=k\times \mathrm{std}\left(I_{\mathrm{map}}\right)+\bar{I_{\mathrm{map}}},\;$$

where $\mathrm{std}\left(*\right)$ represents the standard deviation operation, and k is the segmentation parameter. k has been experimentally proved to show that $k\in \left[10,30\right]$ is effective. When the value of the element in $I_{\mathrm{map}}$ is greater than T, it is set to one, and the opposite is set to zero. The point set to one is the aerial target. The entire procedure of LSM is given in Algorithm 1.
**Algorithm 1** Procedure of LSM.**Input**: Base frame $I_{b}$, reference frames $I_{b-l}$ and $I_{b+l}$. **Output:** The position of the aerial target. (1) Obtain the size $\left[row,col\right]$ of $I_{b}$. (2) **for** x=1:row **do** (3) **for** y=1:col **do**
(4)   Obtain the local slices $R_{11}$ and $R_{2m}$s by Equations (1) and (2); (5)   Obtain the normalized slices $R_{\mathrm{nor}1}$ and $R_{\mathrm{nor}}^{m}$s by Equations (3) and (4); (6)   Calculate the matching coefficient *r*_1_(*x,y*) and determine the $R_{2m_{\max}}$ by Equations (5)–(7); (7)   Construct the spatial–temporal joint model between $I_{b}$ and $I_{b+l}$ and calculate $I_{v1}\left(x,y\right)$ by Equations (8)–(14); (8)   Conduct reverse matching and obtain $R_{31}$ by Equations (15)–(17); (9)   Calculate the normalized slice of $R_{31}$ by Equation (3); (10)  Calculate the matching coefficient $r_{2}\left(x,y\right)$ by Equation (5); (11)  Construct the spatial–temporal joint model between $I_{b-l}$ and $I_{b}$ and calculate $I_{v2}\left(x,y\right)$ by Equations (8)–(14); (12)  Calculate the saliency map value $I_{\mathrm{map}}\left(x,y\right)$ by Equations (18) and (19); (13) **end for**
(14) **end for**
(15) Obtain the saliency map $I_{\mathrm{map}}$; (16) Calculate the adaptive threshold T by formula Equation (20); (17) Output the position of the aerial target.

## 3. Experiments

### 3.1. Experimental Condition and Evaluation Index

The datasets used for experiments were four space-based IR sequences with different backgrounds, and the aerial targets were simulated targets with the same intensity distribution proportions as the real targets. The backgrounds and real targets were obtained from the identical space-based system working under staring imaging mode. The details of the four sequences are found in Table 1. The speeds of aerial targets ranged from 1.1 to 2.0pixel/frame. The value of l was set to two after parameter analysis conducted in Section 4.

To evaluate the detection effectiveness, LSMs are compared with seven state-of-the-art detection methods, including fusion saliency map (FSM) [10], double-neighborhood gradient method (DNGM) [11], neighborhood saliency map (NSM) [14], spatial–temporal local contrast filter (STLCF) [15], spatial–temporal local contrast method (STLCM) [2], spatial–temporal joint processing model (STJP) [28], and multiscale local target characteristics algorithm (MLTC) [29]. NSM, STLCF, STLCM, and STJP are existing space-based detection methods, FSM is a newly proposed detection method utilized for low-altitude slow target detection that has a similar background to space-based detection, and DNGM and MLTC are new detection methods proposed in 2020 and 2021, respectively.

The evaluation indices are background suppression factor (BSF), the gain of signal-to-clutter ratio (GSCR), detection rate ($P_{d}$), false alarm rate ($P_{f}$), and area under the curve (AUC). *BSF* is a global index for evaluating the performance of global background suppression and is defined as:

(21) BSF=σbeforeσafter,

where $\sigma_{\mathrm{after}}$ and $\sigma_{\mathrm{before}}$ are standard deviations of the processed and raw image, respectively. *GSCR* is the index used to evaluate the target enhancement performance. The *GSCR* is calculated by:

(22) SCR=μtar−μbkσbk,

(23) GSCR=SCRafterSCRbefore,

where $\mu_{\mathrm{tar}}$ represents the mean of the target, and $\sigma_{\mathrm{bk}}$ are the mean and standard deviation of the background, respectively, and $SCR_{\mathrm{after}}$ and $SCR_{\mathrm{before}}$ are *SCR* values of the processed and primitive targets, respectively.

The indices used to evaluate the detection effectiveness are $P_{d}$ and $P_{f}$, whose formulas are:

(24) Pd=NdetectedNreal,

(25) Pf=NfalseNpixel,

where $N_{\mathrm{detected}}$ is the number of real targets detected by the method, $N_{\mathrm{real}}$ is the total number of real targets, $N_{\mathrm{false}}$ is the number of targets falsely detected, and $N_{\mathrm{pixel}}$ is the number of pixels. To visualize the detection effectiveness, a receiver operating characteristic curve (ROC) was drawn according to the relationship between $P_{d}$ and $P_{f}$. The area under the ROC curve is represented by AUC.

### 3.2. Experimental Results

**Figure 6:**
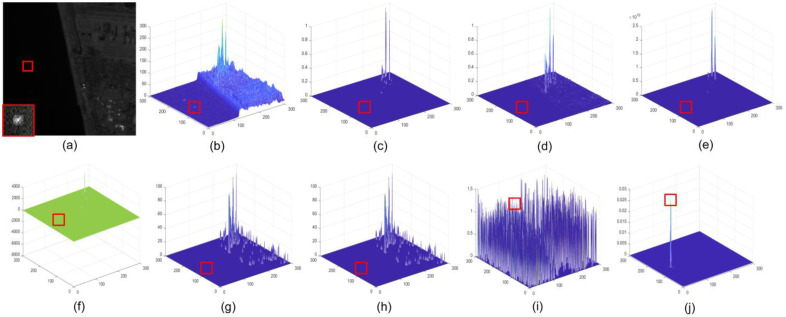
Detection results of different methods in Seq.1. The positions of aerial targets are highlighted by the red rectangles; (**a**) raw image, the details of target are magnified at the left bottom corner; (**b**) three-view of raw image; (**c**) result of STLCM; (**d**) result of STLCF; (**e**) result of FSM; (**f**) result of NSM; (**g**) result of STJP; (**h**) result of DNGM; (**i**) result of MLTC; (**j**) result of LSM.

**Figure 7:**
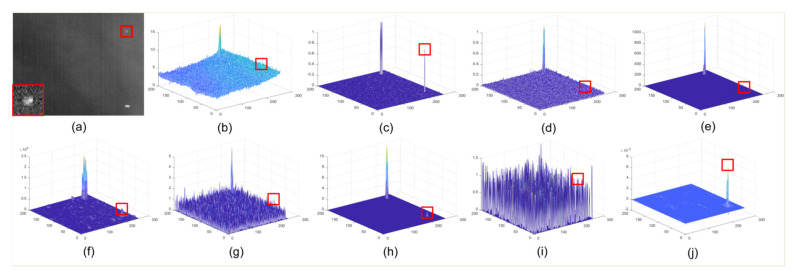
Detection results of different methods in Seq.2. (**a**) raw image, the details of target are magnified at the left bottom corner; (**b**) three-view of raw image; (**c**) result of STLCM; (**d**) result of STLCF; (**e**) result of FSM; (**f**) result of NSM; (**g**) result of STJP; (**h**) result of DNGM; (**i**) result of MLTC; (**j**) result of LSM.

**Figure 8:**
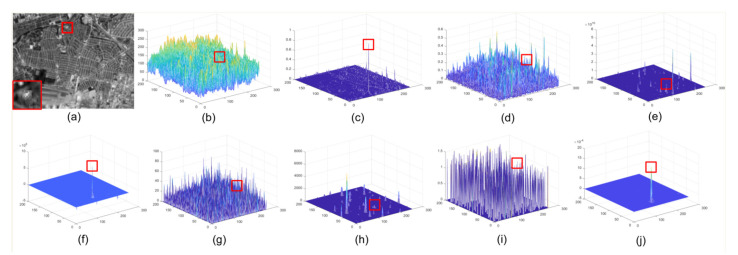
Detection results of different methods in Seq.3. (**a**) raw image, the details of target are magnified at the left bottom corner; (**b**) three-view of raw image; (**c**) result of STLCM; (**d**) result of STLCF; (**e**) result of FSM; (**f**) result of NSM; (**g**) result of STJP; (**h**) result of DNGM; (**i**) result of MLTC; (**j**) result of LSM.

**Figure 9:**
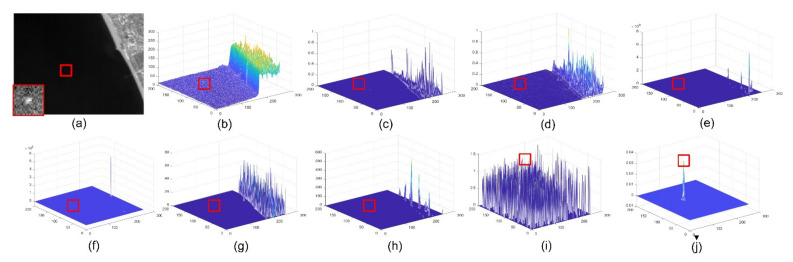
Detection results of different methods in Seq.4. (**a**) raw image, the details of target are magnified at the left bottom corner; (**b**) three-view of raw image; (**c**) result of STLCM; (**d**) result of STLCF; (**e**) result of FSM; (**f**) result of NSM; (**g**) result of STJP; (**h**) result of DNGM; (**i**) result of MLTC; (**j**) result of LSM.

The three views of the results corresponding to different methods are given in Figure 6, Figure 7, Figure 8 and Figure 9. The experimental results under two situations are both exhibited. Under the first situation where the aerial target is much weaker than clutter, as shown in images in Seqs.1, 2, and 4, STLCF, STLCM, FSM, NSM, and DNGM suppress most of the background in the images, but the clutter with strong intensities is still retained. STJP is sensitive to clutter. Only MLTC and our method can enhance the weak targets. However, MLTC still enhances the background component, which results in false alarms. The images in Seq.3 show a situation in which the intensity of a small target is close to strong clutter. Most methods, such as STLCF, STLCM, STJP, MLTC, and the proposed method, perform well on target enhancement, but STLCM, STJP, and MLTC also bring a great number of false alarms. According to the above comparison, our method fits the task to suppress the strong clutter and enhance the weak target simultaneously. The quantitative comparison and analysis are given in the next part, for more compelling results.

The results of *BSF* are listed in Table 2. LSM achieved the highest *BSF* value on Seq.4 but had lower values than FSM or STLCM on the other three sequences because of the zero-setting operation in the two methods. In FSM, when the mean of the variance difference between the internal window and the external window is less than zero, the spatial variance saliency map value of a pixel will be zero, and the output will be zero eventually. In STLCM, the final value of a pixel will be set to zeros if this pixel is not the local maximum point. In an IR image, the local maximum points are usually composed of the small target, clutter, and noise. Therefore, because of the zero-setting operation, those pixels around the local maximum point will be assigned to values of zero. It is clear that the more zero points the final saliency map has, the lower the value of $\sigma_{\mathrm{after}}$ in Equation (21) will be, and the value of *BSF* will consequently increase.

It is worth noting that *BSF* is a global index evaluating the background suppression ability of a method in the whole image, but clutter only accounts for a little proportion in the background. Thus, taking the results in Figure 6, Figure 7, Figure 8 and Figure 9 and Table 2 into consideration, STLCM and FSM fail to suppress the clutter even if they suppress conventional background suppression better than our method. The other methods received lower values than LSM on all sequences. These results indicate that most methods can suppress most backgrounds but have poor abilities to suppress the strong clutter on the space-based IR images. STLCM and STLCF suppress background by the direct interframe difference, in which the weak target is suppressed but residual clutter still exists. MLTC, NSM, and DNGM fail to suppress clutter since it has a similar spatial distribution as the aerial target.

The average *GSCR* values are listed in Table 3. LSM receives the best results on Seqs.1, 3, and 4. STJP and STLCF find it hard to enhance the target in the space-based IR image. STLCM, FSM, and NSM receive malfunctions on Seqs.1, 2, and 4 because of the considerable intensity difference between target and clutter. Only DNGM shows a better result than LSM on Seq.3 but enhances clutter better, as shown in Figure 7. MLTC can enhance the aerial targets that are much weaker than clutter in space-based images, but our method performs better than MLTC, as shown in Table 3.

**Figure 10:**
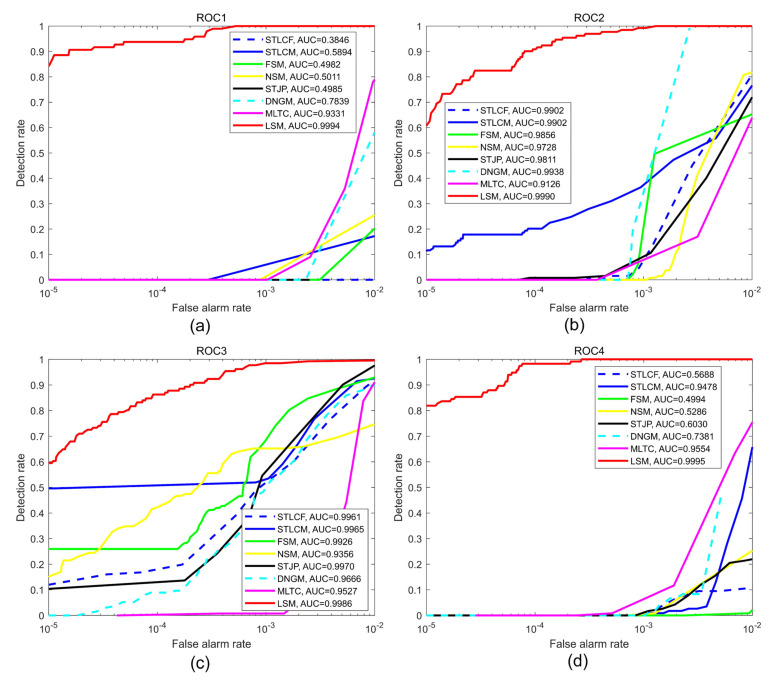
ROC curves and AUC values of different methods for the four sequences; (**a**–**d**) represent Seqs.1–4, respectively.

The results of detection effectiveness are shown by the ROC curves and AUC values in Figure 10 and Table 4. The $P_{d}$s of LSM are more than 85% on the four sequences when $P_{f}$s are $10^{-4}$ and more than 97% when s are $10^{-3}$. The AUC values of LSM on the four sequences are 0.9994, 0.9990, 0.9986, and 0.9995, respectively. However, the results of other methods are unstable on the four sequences. In Figure 10a,d, most AUC values of the most compared methods are less than 0.8 because Seqs.1 and 4 have backgrounds of sea and land, and the intensity of clutter is at least 10 times stronger than that of the target. On Seq.2, DNGM has an AUC value of more than 0.99 because it enhances the target better than LSM. However, the effectiveness of DNGM drops sharply when $P_{f}<10^{-4}$ because DNGM enhances clutter better than target, and MLTC does the same on four sequences. On Seqs.3, the compared methods achieved better detection performance; the five methods obtained AUC values of more than 0.99 because the intensities of the target are close to those of clutter. The average AUC values are also calculated and given in Table 4; MLTC receives the highest average value of 0.9534 because of great target enhancement ability, while the minimum value is 0.7345 belonging to the single-frame method NSM. Compared with the seven methods, the average AUC value of LSM is 0.9991, indicating that LSM has the best detection effectiveness.

According to the experimental results, LSM performs better than the seven compared methods and can detect the aerial targets more effectively on the space-based IR sequences with different backgrounds. The results prove that LSM can conquer the challenge of enhancing the weak target and suppressing the strong clutter simultaneously.

## 4. Analysis and Discussion

Seqs.1–3 contain 7×7 targets and Seq.4 contains 5×5 targets. According to the results, our method enhanced both targets significantly. The *GSCR* values in Table 3 revealed that our method enhanced the targets with different sizes considerably. As for the detection effectiveness, our method obtained $P_{d}>98\%$ when the $P_{f}$s reached $10^{-3}$ on all sequences. Meanwhile, the values of AUC were more than 0.9986. The above results indicate our method can maintain its effectiveness when detecting targets of different sizes.

**Figure 11:**
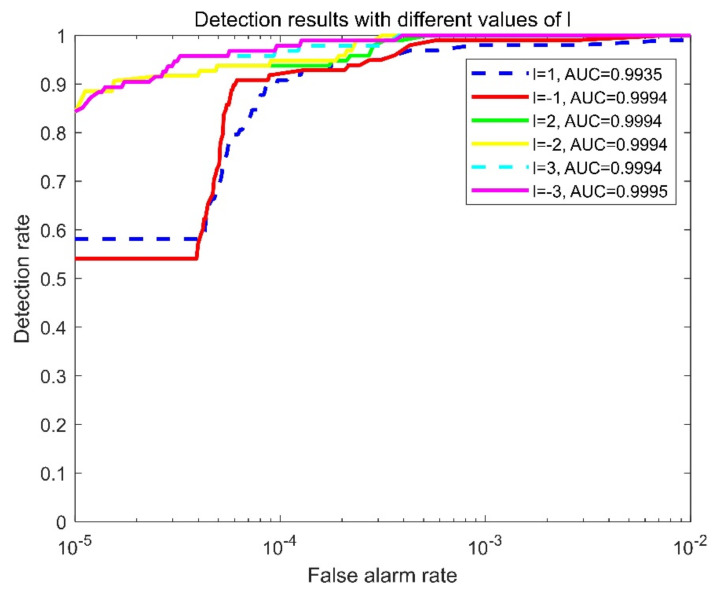
ROCs and AUC values with different frame intervals.

In order to analyze the influence of l, we selected Seq.1 as the example with which to explore the influence of parameter l, in which the target speed is 1.55 pixel/frame; the experiments were conducted with different values of l. Results of $\bar{BSF}$ and $\bar{GSCR}$ are given in Table 5, and the detection effectiveness is shown in Figure 11. The results indicate that there are few evidently different results between l and −l but that the tendencies of *BSF* and *GSCR* are different with $\left|l\right|$ increase. With $\left|l\right|$ increase, the matching coefficient $r_{1}$ decreases, but the dipole is clearer; thus, the target can be further enhanced, but the background cannot be well suppressed. The detection effectiveness shown in Figure 11 reveals that it generates similar detection results when l=±2 or ±3, which is better than the result when l=±1. Therefore, the value of l is recommended to be ±2, and set to two in this paper.

**Figure 12:**
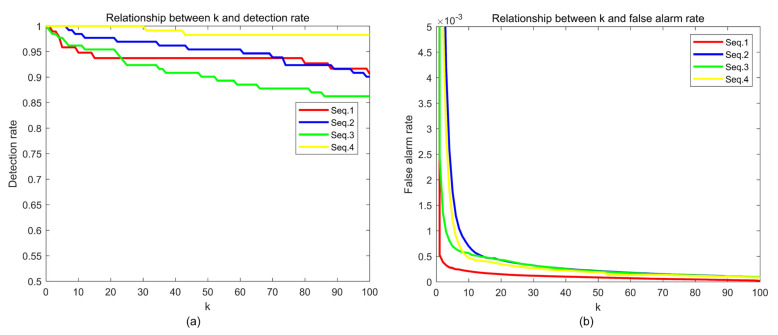
Influence of k. (**a**) Relationship between k and $P_{d}$; (**b**) relationship between *k* and $P_{f}$.

The segmentation parameter k directly influences the detection effectiveness; the relationships between k and detection results are shown in Figure 12. With the increase in the k, both detection rate and false alarm rate decrease. The detection rate of different experimental sequences showed similar trends, and the detection rates could be maintained above 90% when 10≤k≤30. The variation trends of the false alarm rates of the five test sequences are nearly the same as well. When k≥10, the false alarm rates of all the sequences are less than $10^{-3}$. In order to maintain the detection rate ≥90% and false alarm rate $\leq 0.5\times 10^{-3}$, the value range of k in this method is recommended to be $\left[10,30\right]$, which was given in Section 2.5.

## 5. Conclusions

This paper proposes a concise method, which is based on local spatial–temporal matching, for detecting an aerial target on a space-based IR platform. The experimental results determine that, compared with existing methods, LSM exhibits better detection performance when the clutter is much stronger than the aerial target. However, LSM is currently only suitable for the staring imaging mode and still needs to be optimized to adapt to other modes.

## Figures and Tables

**Table 1:** Details of experimental datasets.

Dataset	Size	Target Size	Details of Backgrounds
	Frame		
Seq.1	300×300	7×7	Sea and land background, strong clutter, background moving speed is 0.11 pixel/frame;
	100		
Seq.2	200×256	7×7	Sea background, strong clutter, residual nonuniformity stripe, background moving speed is 0.14 pixel/frame;
	135		
Seq.3	200×256	7×7	Land background, strong clutter, background moving speed is 0.53 pixel/frame;
	135		
Seq.4	200×256	5×5	Sea and land background, strong clutter, background moving speed is 0.24 pixel/frame.
	120		

**Table 2:** Average *BSF* values of different methods for four sequences.

Methods	Seq.1	Seq.2	Seq.3	Seq.4
STLCF	7.0835	3.7508	2.4573	4.2582
STLCM	**14.9534**	**8.3428**	13.0866	13.7626
FSM	11.4422	7.3382	11.1237	**28.0876**
NSM	9.0671	2.4982	13.9567	20.3182
STJP	5.7969	1.1265	1.9641	4.8315
DNGM	6.0336	4.1818	6.2833	14.9458
MLTC	1.2551	0.6357	1.5488	2.1215
Proposed	9.5974	6.7767	**14.2828**	24.0300

**Table 3:** Average *GSCR* values of different methods for four sequences.

Methods	Seq.1	Seq.2	Seq.3	Seq.4
$\bar{SCR_{\mathrm{before}}}$	2.9400	1.7764	2.4502	2.2381
STLCF	1.7490	0.7818	0.6588	0.0897
STLCM	1.7231	2.8566	2.0138	0.5797
FSM	1.7213	1.3366	5.9280	0.0028
NSM	0.0118	0.7149	2.1257	0.0269
STJP	0.6507	0.1349	0.4681	0.0795
DNGM	0.7587	**12.8578**	7.0548	0.4568
MLTC	13.1471	1.2697	8.7553	28.2292
Proposed	**17.5890**	7.4260	**11.7774**	**28.4823**

**Table 4:** AUC values of different methods. The last column shows the average values.

Methods	Seq.1	Seq.2	Seq.3	Seq.4	Average
STLCF	0.3846	0.9902	0.9961	0.5688	0.7350
STLCM	0.5894	0.9902	0.9965	0.9478	0.8809
FSM	0.4982	0.9856	0.9926	0.4994	0.7440
NSM	0.5011	0.9728	0.9356	0.5286	0.7345
STJP	0.4985	0.9811	0.9970	0.6030	0.7699
DNGM	0.7839	0.9938	0.9666	0.7381	0.8706
MLTC	0.9331	0.9126	0.9527	0.9554	0.9534
Proposed	**0.9994**	**0.9990**	**0.9986**	**0.9995**	**0.9991**

**Table 5:** The values of *GSCR* and *BSF* with different frame intervals.

l	−3	−2	−1	1	2	3
$\bar{GSCR}$	24.7905	17.2655	10.7153	10.7153	17.5890	24.7880
$\bar{BSF}$	9.3730	9.4456	9.5933	9.5933	9.5974	9.3730

## Data Availability

Not applicable.
